# Cholangioscopy-assisted ERCP reduces radiation exposure in treating choledocholithiasis: a retrospective IPTW-adjusted cohort study

**DOI:** 10.3389/fmed.2026.1816788

**Published:** 2026-07-03

**Authors:** Junpu Wang, Zifeng Huang, Pengwei Zhang, Yihuang Lin, Guoqing Huang, Fan Jiang, Shi Qiu

**Affiliations:** Gallstone Disease Center, The Affiliated Puren Hospital of Wuhan University of Science and Technology, Wuhan, China

**Keywords:** cholangioscopy-assisted ERCP, choledocholithiasis, common bile duct stones, endoscopic retrograde cholangiopancreatography, fluoroscopy, radiation dose

## Abstract

**Background:**

Conventional ERCP for common bile duct stones relies on fluoroscopy, which raises concerns about radiation exposure and may not fully exclude residual stones. This study compared cholangioscopy-assisted ERCP with conventional fluoroscopy-guided ERCP in routine treatment of choledocholithiasis.

**Methods:**

This single-center retrospective study included 100 patients with bile duct stones treated between June 2024 and June 2025. The cholangioscopy-assisted group included patients who underwent electronic mother-baby cholangioscopy-assisted ERCP (*n* = 50), and the conventional group was selected from contemporaneous conventional fluoroscopy-guided ERCP cases using age-stratified random sampling (*n* = 50). The primary endpoint was the intra-procedural radiation-dose reading measured in mGy by an RG1000 radiation dose meter placed at the routine patient position. Secondary outcomes included procedure time, duct clearance, adverse events, length of stay, and hospitalization cost. Confounding was addressed using stabilized inverse probability of treatment weighting (IPTW).

**Results:**

Median radiation-dose reading was lower in the cholangioscopy-assisted group than in the conventional group (9.93 vs. 14.64 mGy, *p* < 0.001). After IPTW, cholangioscopy assistance remained associated with a reduced radiation-dose reading (geometric mean ratio [GMR] 0.687, 95% CI 0.610–0.774; *p* < 0.001) and a lower risk of high radiation exposure (risk ratio 0.301, 95% CI 0.095–0.955; *p* = 0.042). Procedure time and hospitalization cost were higher, whereas length of stay was shorter. Complete clearance in one session was 88.0% versus 94.0%, and all patients requiring second-session ERCP achieved final duct clearance. All post-ERCP pancreatitis cases were mild, and no statistically significant difference in short-term adverse events was observed between groups.

**Conclusion:**

Cholangioscopy-assisted ERCP was associated with lower intra-procedural radiation-dose reading and fewer high-dose procedures in patients treated for choledocholithiasis. No statistically significant difference in short-term adverse events was observed, but longer procedure duration and higher cost were noted. These findings should be confirmed in prospective studies using standardized radiation metrics and longer follow-up.

## 1 Introduction

Common bile duct stones (CBDS) complicate gallstone disease in approximately 5–15% of patients and can precipitate acute cholangitis, pancreatitis, and obstructive jaundice. Endoscopic retrograde cholangiopancreatography (ERCP) remains the cornerstone intervention for duct clearance and is recommended by major society guidelines (1, 2). However, ERCP is not a benign procedure and carries a clinically meaningful adverse-event burden, underscoring the importance of achieving complete duct clearance efficiently and safely within a single session (2).

In routine practice, bile duct clearance is commonly assessed by balloon-occlusion cholangiography after stone extraction. Nevertheless, occlusion cholangiography has known blind spots and may miss residual stones or fragments, particularly in patients with a dilated bile duct and/or those undergoing lithotripsy (3). In a prospective tandem study, digital cholangioscopy detected residual biliary stones despite a negative occlusion cholangiogram in a substantial proportion of cases (3). Consistently, a recent systematic review and meta-analysis suggested that residual stones may remain after apparently complete clearance and can be uncovered by cholangioscopy, highlighting a clinically relevant gap in conventional confirmation strategies (4). In parallel, recurrent bile duct stones after endoscopic clearance remain a non-negligible problem at the population level; a systematic review reported that recurrence occurs in approximately one out of seven patients, emphasizing the need to optimize clearance verification and risk-stratified follow-up pathways (5).

ERCP is fluoroscopy-dependent, raising concerns regarding ionizing radiation exposure for both patients and endoscopy staff. Multi-centre data confirm substantial inter-procedure and inter-institution variability in patient radiation dose during ERCP, supporting ongoing quality-improvement efforts to standardize and reduce exposure (6). Occupational dosimetry studies further demonstrate procedure-specific exposure patterns for operators and assistants (7) and emphasize that eye-lens exposure is an important safety consideration in ERCP rooms (8). Contemporary guidance from gastroenterology societies and international Delphi consensus work has therefore increasingly focused on practical fluoroscopy standards and dose-reduction strategies (e.g., pulsed fluoroscopy, collimation, last-image hold, appropriate shielding, and real-time dose awareness) to operationalize the ALARA principle in everyday endoscopy practice (9, 10). A prospective randomized study also showed that low-dose pulsed fluoroscopy can substantially reduce radiation exposure during ERCP without compromising perceived image quality or procedural efficiency, reinforcing the feasibility of dose-optimization in routine workflows (11).

Cholangioscopy has evolved from a niche modality to a technique with expanding indications, supported by recent clinical practice guidelines and training/quality standards (12, 13). Beyond enabling targeted lithotripsy and biopsy under direct vision, cholangioscopy-assisted ERCP provides a plausible pathway to improve duct-clearance verification and, in selected settings, to reduce reliance on fluoroscopy. Early multicenter experience has also suggested feasibility of fluoroscopy-free direct solitary cholangioscopy for non-complex bile duct stones, implying that direct-visualization workflows may offer both clinical and radiological advantages when appropriately implemented (14). Nevertheless, real-world evidence remains limited regarding whether incorporating cholangioscopy into routine ERCP pathways for CBDS can meaningfully reduce fluoroscopy exposure while maintaining short-term clinical outcomes, particularly in resource-constrained settings where procedural efficiency and cost considerations are highly relevant.

Accordingly, we conducted a retrospective comparative study to evaluate whether cholangioscopy-assisted ERCP, compared with conventional fluoroscopy-guided ERCP, is associated with reduced fluoroscopy exposure in patients undergoing endoscopic treatment for CBDS. We further assessed short-term clinical outcomes, adverse events, length of stay, and cost to inform pragmatic adoption of cholangioscopy within routine stone-management pathways.

## 2 Methods

### 2.1 Study design and reporting

This single-center retrospective comparative study evaluated cholangioscopy-assisted ERCP versus conventional fluoroscopy-guided ERCP for the treatment of common bile duct (CBD) stones. The primary outcome was the intra-procedural radiation-dose reading, and secondary outcomes included technical and clinical outcomes, procedure time, length of stay, hospitalization cost, and short-term adverse events. The study was reported in accordance with the STROBE statement for observational studies.

### 2.2 Setting, eligibility, and group allocation

Adult patients who underwent ERCP for CBD stones at the Gallstone Disease Center, the Affiliated Puren Hospital of Wuhan University of Science and Technology, between June 2024 and June 2025 were screened. Eligible patients were aged 18 to 85 years and had CBD stones confirmed by pre-procedural imaging, including abdominal computed tomography, ultrasonography, or magnetic resonance imaging/magnetic resonance cholangiopancreatography. Patients were eligible when the largest stone diameter was no more than 2.0 cm, the number of stones was no more than five, and complete peri-procedural and follow-up records were available.

The exclusion criteria were: (1) upper gastrointestinal stricture or obstruction precluding duodenoscope access to the second portion of the duodenum; (2) severe cardiopulmonary dysfunction or other conditions that made the patient unable to tolerate endoscopic treatment; (3) clinically significant coagulopathy, malignant biliary stricture, or suspected or confirmed ampullary or biliary malignancy; and (4) acute obstructive suppurative cholangitis or septic shock.

Patients in the cholangioscopy-assisted group were those who underwent ERCP with electronic mother-baby cholangioscopy during the study period. Patients in the conventional group were selected from contemporaneous conventional fluoroscopy-guided ERCP cases using age-stratified random sampling. The use of cholangioscopy was determined jointly by patient preference and the treating endoscopist’s clinical judgment. Therefore, group allocation was not randomized, and potential selection bias was addressed using propensity score-based methods and acknowledged in the interpretation of the findings.

### 2.3 Devices, operators, and radiation-dose measurement

All procedures were performed by the same experienced ERCP team, consisting of board-certified endoscopists with independent ERCP practice experience. Key devices included an Olympus duodenoscope and a 9-Fr electronic mother-baby cholangioscopy system (eyeMAX Dongcha; Micro-Tech, Nanjing, China), together with standard ERCP accessories, including sphincterotomes, guidewires, dilation balloons, extraction balloons, extraction baskets, nasobiliary drains, biliary stents, and pancreatic duct stents.

The intra-procedural radiation-dose reading was recorded in mGy using an RG1000 radiation dose alarm meter (smaach, RG1000), which had been certified by the China North National Center of Metrology and Testing. The meter was placed at the routine patient position to record the dose reading during the procedure. The meter was reset after each procedure and calibrated every 3 months. Fluoroscopy was performed using an X-ray system from Beijing General Electric Hualun Medical Equipment Co., Ltd. (REF: 5450683-03; SN: H2821365). Fluoroscopy time, cumulative air kerma, and dose-area product were not routinely exported or recorded during the study period and were therefore unavailable.

### 2.4 ERCP procedures

#### 2.4.1 Conventional fluoroscopy-guided ERCP

With the patient in the semi-prone position, the duodenoscope was advanced to the second portion of the duodenum to locate the major papilla. Rectal diclofenac was administered before ERCP for post-ERCP pancreatitis prophylaxis unless contraindicated. Biliary cannulation was performed using a sphincterotome and guidewire. After successful access, a small amount of contrast was injected to delineate the biliary and pancreatic ducts under fluoroscopy. For difficult cannulation, adjunctive techniques such as precut sphincterotomy or the double-guidewire technique were used when needed. If pancreatic duct cannulation occurred more than three times, the double-guidewire technique and prophylactic pancreatic duct stenting were considered according to the endoscopist’s judgment.

Endoscopic sphincterotomy and/or papillary balloon dilation was performed according to papillary status, prior sphincterotomy history, stone size, and ductal anatomy. Stone extraction was performed using an extraction balloon or basket, and lithotripsy was used when required. After extraction, balloon-occlusion cholangiography was performed to assess residual filling defects. Nasobiliary drainage or biliary stenting was used as part of the center’s routine post-ERCP management pathway when clinically indicated, mainly for biliary drainage, observation of bile characteristics, reduction of cholangitis risk, and post-procedural management; the final decision also considered patient or authorized representative preference.

#### 2.4.2 Cholangioscopy-assisted ERCP

Initial steps were identical to those in the conventional group, including selective biliary cannulation and an initial fluoroscopic cholangiogram to confirm ductal anatomy and approximate stone burden. Subsequently, the electronic mother-baby cholangioscope was introduced through the working channel of the duodenoscope. Under direct visualization, the CBD and accessible intrahepatic branches were systematically inspected to define stone size, number, and distribution, and the bile duct mucosa was evaluated for stricture or neoplasm. Targeted biopsy was performed when needed.

Stone extraction was then performed using a suitable basket, balloon, or other device according to the direct visual findings. After extraction, cholangioscopy was performed again to confirm the absence of residual stones and obvious mucosal abnormalities. Nasobiliary drainage or biliary stenting was placed according to the same clinical principles as in the conventional group.

### 2.5 Outcomes and data collection

Data were extracted from electronic medical records and procedure records. Baseline and disease-related variables included age, sex, body mass index, hypertension, diabetes, coronary heart disease, prior cholecystectomy, prior ERCP, prior endoscopic sphincterotomy, American Society of Anesthesiologists physical status, number of stones, largest stone diameter, CBD diameter, stone location, total and direct bilirubin levels, and presence of cholangitis. Procedural variables included procedure time, difficult cannulation, precut sphincterotomy, double-guidewire technique, pancreatic duct opacification, endoscopic sphincterotomy, papillary balloon dilation, lithotripsy, extraction method, nasobiliary drainage, biliary stent placement, and pancreatic duct stent placement.

Clinical outcomes included successful biliary cannulation, successful stone extraction, complete clearance in one session, final duct clearance after second-session ERCP when required, post-ERCP pancreatitis, post-ERCP bleeding, length of stay, and total hospitalization cost. Total hospitalization cost included all costs incurred from admission to discharge, including the cost of electronic mother-baby cholangioscopy and related accessories when used. The discharge criteria after ERCP were stable vital signs, absence of clinically relevant abdominal pain, fever, nausea, or vomiting, near-normalization or clear improvement of laboratory indices such as serum amylase, liver function tests, and blood counts, ability to ambulate freely, and tolerance of oral intake. Nasobiliary drainage was removed when the patient had no severe complications and follow-up laboratory tests were clinically acceptable.

The primary radiation endpoint was the intra-procedural radiation-dose reading in mGy recorded by the RG1000 meter placed at the routine patient position. Because fluoroscopy time, cumulative air kerma, and dose-area product were not recorded, these complementary radiation metrics could not be analyzed.

### 2.6 Definitions of adverse events and duct clearance

Post-ERCP pancreatitis was defined and graded according to widely used consensus criteria referenced in the ASGE guideline on ERCP adverse events, requiring new or worsened pancreatic-type abdominal pain with pancreatic enzyme elevation of at least three times the upper limit of normal after ERCP and unplanned hospitalization or prolongation of admission. Severity was categorized according to hospitalization duration and the presence of complications or need for intervention. All post-ERCP pancreatitis cases in this cohort were mild and resolved after conservative treatment, including fasting, intravenous fluid therapy, acid suppression, enzyme inhibition, and monitoring of serum amylase at 3 and 24 h after ERCP. Post-ERCP bleeding was identified according to standard ERCP adverse-event definitions, including clinical evidence of bleeding with a relevant hemoglobin decrease and/or need for endoscopic, radiologic, or transfusion intervention.

Complete clearance in one session was defined as absence of residual stones at the end of the index ERCP session, as confirmed by balloon-occlusion cholangiography in the conventional group or repeat direct cholangioscopic inspection in the cholangioscopy-assisted group. Final duct clearance was defined as complete clearance after the index procedure or after second-session ERCP when the index procedure was terminated early or complete clearance could not be achieved in one session.

### 2.7 Statistical analysis

Continuous variables were assessed for normality using the Shapiro–Wilk test. Normally distributed variables were summarized as mean ± standard deviation and compared using the independent-samples *t* test. Non-normally distributed variables were summarized as median and interquartile range and compared using the Mann–Whitney U test. Categorical variables were summarized as *n* (%) and compared using the chi-square test or Fisher’s exact test when expected counts were small. Two-sided *p* values less than 0.05 were considered statistically significant.

Because of the non-randomized retrospective design, stabilized inverse probability of treatment weighting (IPTW) based on propensity scores was performed to reduce measured confounding. Propensity scores were estimated using logistic regression with prespecified baseline and pre-procedural disease-related covariates, including age, sex, body mass index, hypertension, diabetes, coronary heart disease, prior cholecystectomy, having two or more stones, largest stone diameter, CBD diameter, total bilirubin level, cholangitis, and American Society of Anesthesiologists physical status. Covariate balance before and after weighting was assessed using standardized mean differences, with an absolute standardized mean difference less than 0.10 considered acceptable. Balance was also visualized using a Love plot, and overlap of propensity scores was evaluated graphically.

For IPTW-adjusted analyses of skewed continuous outcomes, including radiation-dose reading, procedure time, length of stay, and total hospitalization cost, weighted log-linear models were fitted, and exponentiated coefficients were reported as geometric mean ratios with 95% confidence intervals. The primary radiation analysis used the radiation-dose reading as a continuous variable. High radiation exposure was also evaluated as an exploratory binary endpoint using the 75th percentile of the conventional group as the threshold, and relative risks were estimated using Poisson regression with robust variance in both unweighted and IPTW-weighted analyses. Spearman correlation was used to evaluate the association between procedure time and radiation dose within each group. Conventional multivariable regression was used as a sensitivity analysis. All statistical analyses were performed using IBM SPSS Statistics, version 31.0.1.0 (IBM Corp., Armonk, NY, United States).

## 3 Results

### 3.1 Baseline characteristics

A total of 100 patients with common bile duct stones were included, with 50 patients in the cholangioscopy-assisted group and 50 in the conventional fluoroscopy-guided group. Baseline and disease-related characteristics are summarized in Table 1. The cholangioscopy-assisted group was numerically older than the conventional group, but the difference was not statistically significant (median 68.50 vs. 62.00 years; *p* = 0.065). Most demographic and disease-related variables were comparable between groups, including BMI, maximum stone diameter, CBD diameter, cholangitis, prior ERCP, prior EST, altered anatomy, ASA score, and stone location. Total bilirubin and direct bilirubin were lower in the cholangioscopy-assisted group than in the conventional group (*p* = 0.032 and *p* = 0.036, respectively); total bilirubin was included in the propensity score model, and the bilirubin imbalance was considered when interpreting the adjusted results.

**Table 1:** Baseline and disease-related characteristics.

Characteristic	Cholangioscopy-assisted (*n* = 50)	Conventional (*n* = 50)	*P* value
Age, years	68.50 (59.00, 75.00)	62.00 (55.00, 71.00)	0.065
Male sex	21/50 (42.0%)	23/50 (46.0%)	0.687
BMI, kg/m^2^	24.07 (21.98, 27.11)	24.21 (22.01, 26.61)	0.710
Hypertension	20/50 (40.0%)	21/50 (42.0%)	0.839
Diabetes	10/50 (20.0%)	14/50 (28.0%)	0.349
Coronary heart disease	7/50 (14.0%)	9/50 (18.0%)	0.585
Prior cholecystectomy	39/50 (78.0%)	33/50 (66.0%)	0.181
Maximum stone diameter, mm	8.00 (5.00, 10.00)	6.00 (5.25, 8.00)	0.289
CBD diameter, mm	15.00 (12.00, 18.00)	13.00 (12.00, 15.00)	0.082
Total bilirubin, μmol/L	23.00 (9.77, 43.19)	33.90 (18.83, 64.20)	0.032
Direct bilirubin, μmol/L	11.00 (3.95, 27.85)	19.40 (8.68, 45.08)	0.036
≥2 stones	33/50 (66.0%)	26/50 (52.0%)	0.155
Cholangitis	33/50 (66.0%)	32/50 (64.0%)	0.834
Prior ERCP	4/50 (8.0%)	1/50 (2.0%)	0.362
Prior EST	5/50 (10.0%)	1/50 (2.0%)	0.204
Altered anatomy	2/50 (4.0%)	1/50 (2.0%)	1.000
Periampullary diverticulum	17/50 (34.0%)	9/50 (18.0%)	0.068
ASA score I/II/III/IV	1/25/23/1	0/34/16/0	0.201
Stone location distal/middle/proximal/multiple	26/6/1/17	26/4/0/20	0.650

### 3.2 Procedural characteristics

**Figure 1:**
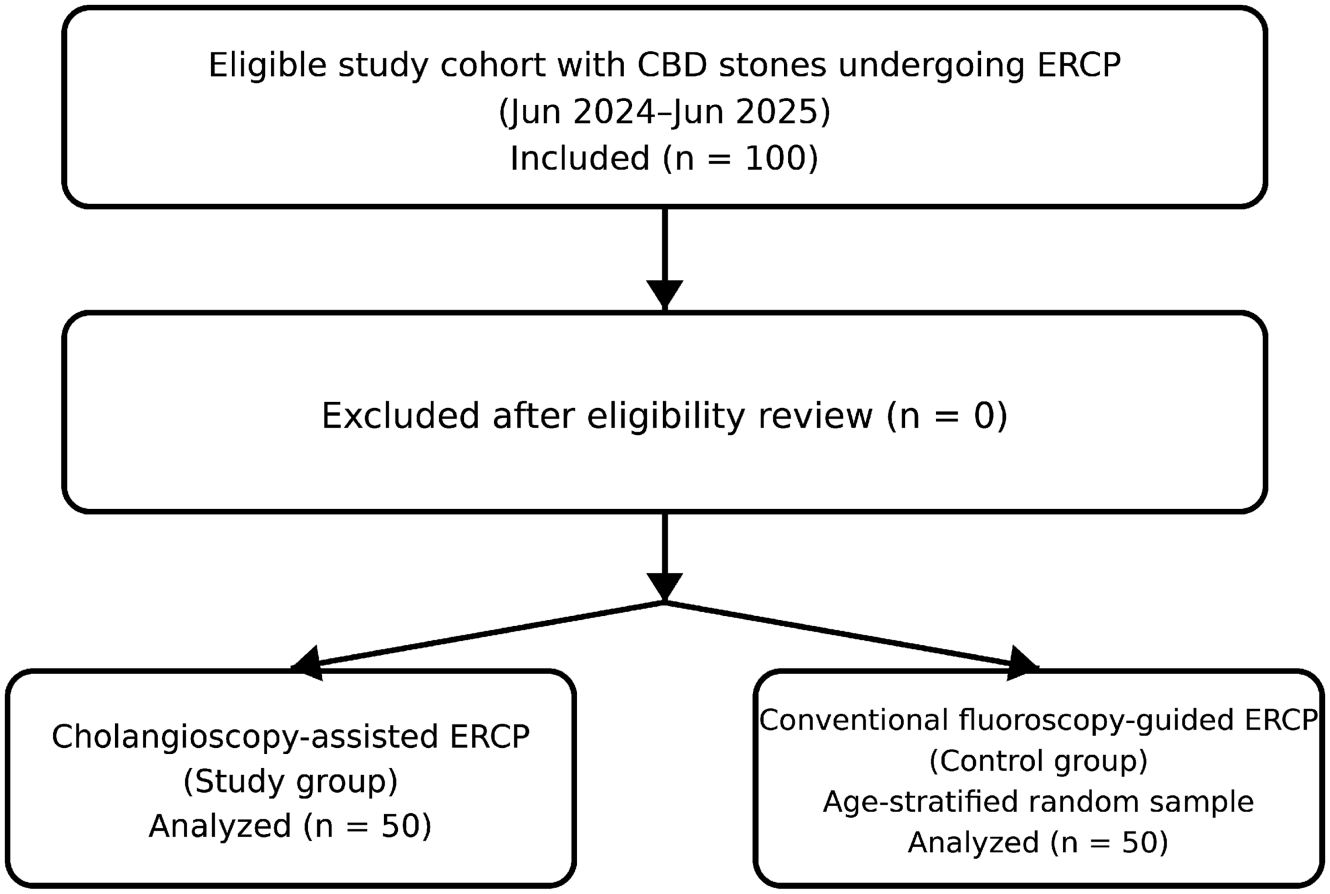
Study flow diagram of patient inclusion and group allocation. CBD, Common bile duct; ERCP, Endoscopic retrograde cholangiopancreatography.

Procedural characteristics are summarized in Table 2. The cholangioscopy-assisted group had a longer procedure time than the conventional group (median 66.00 vs. 50.00 min; *p* < 0.001). The median cholangioscopy inspection time was 10.90 min. Basket use was more frequent in the cholangioscopy-assisted group (76.0% vs. 54.0%; *p* = 0.021), whereas balloon use was less frequent (42.0% vs. 80.0%; *p* < 0.001). The use of EST, nasobiliary drainage, biliary stenting, pancreatic duct stenting, difficult cannulation techniques, precut sphincterotomy, double-guidewire technique, pancreatic duct opacification, papillary balloon dilation, and lithotripsy did not differ significantly between groups (Figure 1).

**Table 2:** Procedural characteristics.

Procedure variables	Cholangioscopy-assisted (*n* = 50)	Conventional (*n* = 50)	*P* value
Procedure time, min	66.00 (50.00, 85.00)	50.00 (40.00, 65.00)	<0.001
Cholangioscopy inspection time, min	10.90 (8.72, 14.52)	Not applicable	
Endoscopic sphincterotomy	45/50 (90.0%)	49/50 (98.0%)	0.204
Basket use	38/50 (76.0%)	27/50 (54.0%)	0.021
Balloon use	21/50 (42.0%)	40/50 (80.0%)	<0.001
Nasobiliary drainage	43/50 (86.0%)	47/50 (94.0%)	0.182
Biliary stent placement	8/50 (16.0%)	4/50 (8.0%)	0.218
Pancreatic duct stent placement	9/50 (18.0%)	8/50 (16.0%)	0.790
Difficult cannulation	2/50 (4.0%)	4/50 (8.0%)	0.678
Precut sphincterotomy	3/50 (6.0%)	2/50 (4.0%)	1.000
Double-guidewire technique	9/50 (18.0%)	7/50 (14.0%)	0.585
Pancreatic duct opacification/entry	10/50 (20.0%)	7/50 (14.0%)	0.424
EPBD/papillary balloon dilation	50/50 (100.0%)	50/50 (100.0%)	1.000
Lithotripsy	5/50 (10.0%)	1/50 (2.0%)	0.204

### 3.3 Clinical outcomes and radiation exposure

**Figure 2:**
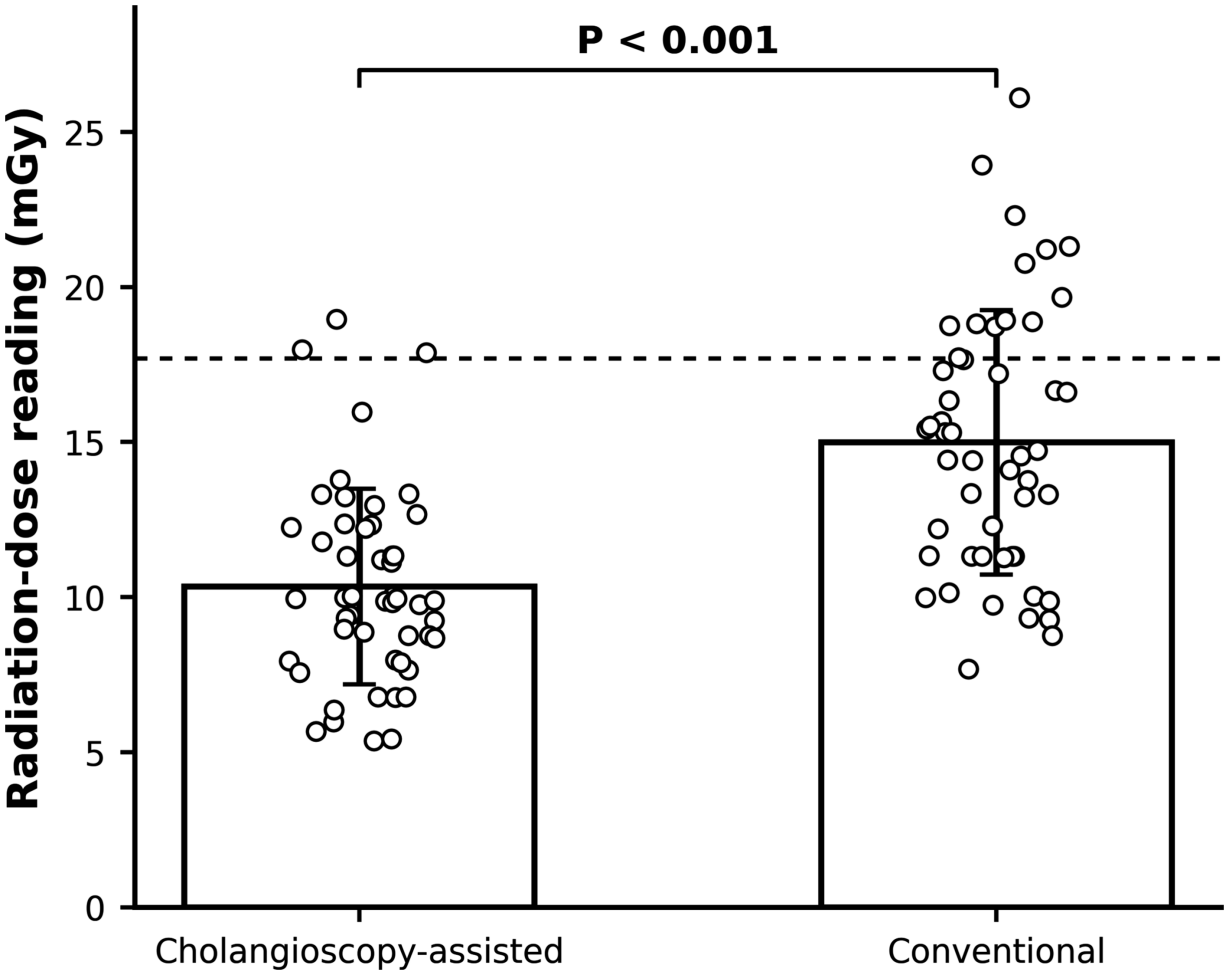
Intraoperative radiation-dose reading by group. Bars indicate mean ± SD, and dots indicate individual patients. The dashed line indicates the 75th percentile of the control group (17.70 mGy), used to define high radiation exposure.

Clinical outcomes and radiation exposure are presented in Table 3. Successful biliary cannulation was achieved in all patients in both groups. Successful stone extraction was achieved in 50/50 patients (100.0%) in the cholangioscopy-assisted group and 48/50 patients (96.0%) in the conventional group (*p* = 0.495). Complete clearance in one session was 88.0% versus 94.0%, respectively (*p* = 0.487). Nine patients without complete clearance during the index ERCP session underwent second-session ERCP, and final duct clearance was achieved in all patients. The cholangioscopy-assisted group had a significantly lower intra-procedural radiation-dose reading than the conventional group (median 9.93 vs. 14.64 mGy; *p* < 0.001). Post-ERCP bleeding occurred in 0% versus 2.0% of patients (*p* = 1.000). PEP occurred in 16.0% versus 22.0% of patients (*p* = 0.444); all cases were mild and resolved after conservative treatment. No patient developed severe pancreatitis, organ failure, required ICU admission, or underwent invasive intervention. The cholangioscopy-assisted group had a shorter length of stay (median 7.00 vs. 9.00 days; *p* < 0.001) but higher total hospitalization cost (median 41,651.32 vs. 31,582.15 CNY; *p* < 0.001) (Figure 2).

**Table 3:** Clinical outcomes and radiation exposure.

Outcome	Cholangioscopy-assisted (*n* = 50)	Conventional (*n* = 50)	*P* value
Intra-procedural radiation-dose reading, mGy	9.93 (8.16, 12.25)	14.64 (11.32, 17.70)	<0.001
Successful cannulation	50/50 (100.0%)	50/50 (100.0%)	1.000
Successful stone extraction	50/50 (100.0%)	48/50 (96.0%)	0.495
Complete clearance in one session	44/50 (88.0%)	47/50 (94.0%)	0.487
Second-session ERCP/re-intervention	6/50 (12.0%)	3/50 (6.0%)	0.487
Final duct clearance	50/50 (100.0%)	50/50 (100.0%)	1.000
High radiation exposure (>17.70 mGy)	3/50 (6.0%)	13/50 (26.0%)	0.012
Post-ERCP bleeding	0/50 (0.0%)	1/50 (2.0%)	1.000
Post-ERCP pancreatitis	8/50 (16.0%)	11/50 (22.0%)	0.444
PEP severity	All mild; resolved conservatively	All mild; resolved conservatively	
Length of stay, days	7.00 (6.00, 8.00)	9.00 (8.00, 10.00)	<0.001
Total hospitalization cost, CNY	41,651.32 (38,190.67, 44,533.68)	31,582.15 (29,858.18, 34,214.95)	<0.001

### 3.4 IPTW-adjusted analyses

**Figure 3:**
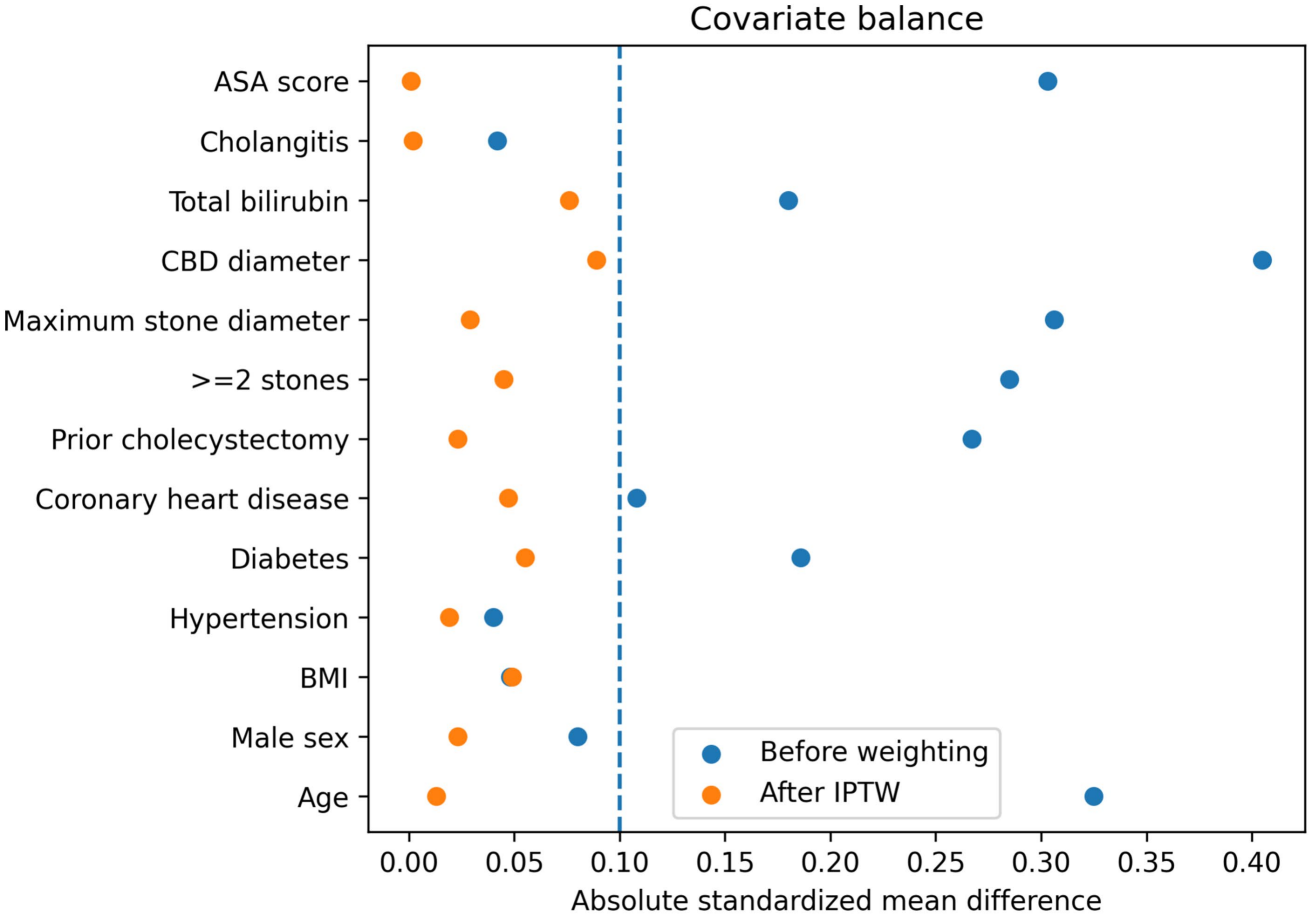
Covariate balance before and after IPTW. The vertical reference line indicates an absolute standardized mean difference of 0.10.

**Figure 4:**
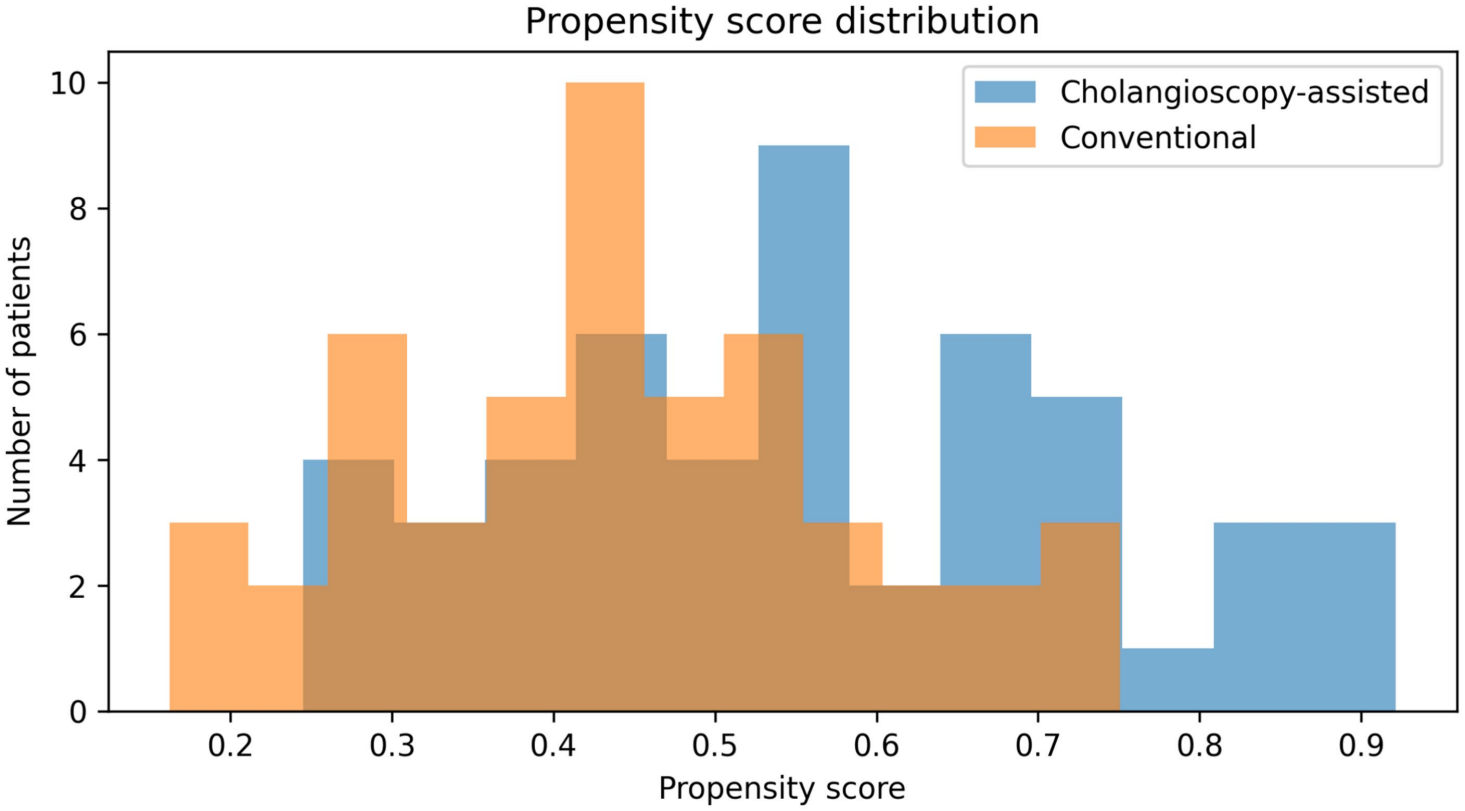
Propensity score distribution by treatment group before IPTW.

To address potential confounding inherent to this retrospective comparison, stabilized inverse probability of treatment weighting (IPTW) based on propensity scores was performed. The propensity score model included age, sex, BMI, hypertension, diabetes, coronary heart disease, prior cholecystectomy, having two or more stones, maximum stone diameter, CBD diameter, total bilirubin, cholangitis, and ASA score. The propensity score range was 0.162–0.921, and the stabilized weight range was 0.543–2.042, with no evidence of extreme weights. Balance improved after weighting, and all covariates included in the propensity score model achieved standardized mean differences below 0.10 after IPTW (Figure 3; Supplementary Table S1). The propensity score distributions before weighting are shown in Figure 4.

### 3.5 High radiation exposure risk

High radiation exposure was defined as an intra-procedural radiation-dose reading above the 75th percentile of the conventional group (17.70 mGy). High exposure occurred in 6.0% (3/50) of the cholangioscopy-assisted group and 26.0% (13/50) of the conventional group. In IPTW-weighted Poisson regression with robust variance, cholangioscopy assistance remained associated with a lower risk of high radiation exposure (RR 0.301, 95% CI 0.095–0.955; *p* = 0.042) (Table 4).

**Table 4:** IPTW-adjusted effect estimates.

Endpoint	Effect measure	IPTW-adjusted effect (95% CI)	*P* value
Intra-procedural radiation-dose reading	GMR	0.687 (0.610–0.774)	<0.001
Procedure time	GMR	1.240 (1.083–1.420)	0.002
Length of stay	GMR	0.793 (0.716–0.879)	<0.001
Total hospitalization cost	GMR	1.272 (1.204–1.343)	<0.001
High radiation exposure (>17.70 mGy)	RR	0.301 (0.095–0.955)	0.042

### 3.6 Association between procedure time and radiation-dose reading

In Spearman correlation analyses, the radiation-dose reading was positively correlated with procedure time in both groups, with a stronger correlation in the conventional group (rho = 0.795, *p* < 0.001) than in the cholangioscopy-assisted group (rho = 0.479, *p* < 0.001). This finding suggests that direct visualization may partially decouple radiation exposure from overall procedure duration, although procedure time remained clinically relevant.

### 3.7 Sensitivity analysis

Sensitivity analysis using an extended multivariable model incorporating additional procedural complexity variables, including difficult cannulation, precut sphincterotomy, double-guidewire technique, pancreatic duct opacification, and lithotripsy, yielded consistent findings for the radiation-dose reading (GMR 0.705, 95% CI 0.611–0.814; *p* < 0.001). These results support the robustness of the primary IPTW analysis, while residual confounding remains possible because of the retrospective non-randomized design.

## 4 Discussion

In this retrospective cohort study, cholangioscopy-assisted ERCP was associated with a lower intra-procedural radiation-dose reading than conventional fluoroscopy-guided ERCP in patients treated for choledocholithiasis. After IPTW adjustment using demographic and disease-related variables, with additional procedural-complexity variables evaluated in sensitivity analysis, cholangioscopy assistance remained associated with a 31.3% lower radiation-dose reading (GMR 0.687) and a lower risk of high radiation exposure (RR 0.301 for >17.70 mGy). These findings suggest that adding direct intraductal visualization may reduce reliance on repeated fluoroscopic assessment, although the observed benefit was accompanied by longer procedure duration and higher hospitalization cost.

A plausible procedural explanation is that conventional ERCP depends on intermittent fluoroscopic checks to define ductal anatomy, localize stones, guide extraction, and confirm clearance. Cholangioscopy permits direct visualization of the bile duct lumen and can therefore reduce the need for repeated cholangiographic confirmation, especially during the final clearance-assessment phase. This interpretation is consistent with previous evidence showing that digital cholangioscopy can detect residual stones after a negative balloon-occlusion cholangiogram (3, 4). In the present cohort, complete clearance in one session was numerically lower in the cholangioscopy-assisted group, but all patients with incomplete index-session clearance underwent second-session ERCP and achieved final duct clearance. The lower one-session clearance rate may partly reflect a stricter endpoint under direct visualization, whereby small residual fragments that might be missed by cholangiography alone were identified rather than overlooked.

The radiation endpoint also requires careful interpretation. The RG1000 meter was placed at the routine patient position and recorded an intra-procedural radiation-dose reading in mGy. Therefore, the primary endpoint reflects the patient-position dose reading captured by an external meter rather than fluoroscopy-system-derived dose-area product, cumulative air kerma, or fluoroscopy time. These complementary radiation metrics were not routinely exported or recorded during the study period. Accordingly, the present findings support a radiation-sparing association at the measured patient position, but they should not be interpreted as a complete dosimetric characterization of patient and staff exposure.

Short-term adverse events should also be interpreted cautiously. Although no statistically significant difference in post-ERCP bleeding or PEP was observed, the sample size was not powered to establish equivalent safety. The observed PEP rates were higher than those commonly expected in unselected choledocholithiasis cohorts, which may relate to strict diagnostic surveillance, including post-procedure amylase monitoring at 3 and 24 h, and the inclusion of hospitalized patients. Importantly, all PEP events in both groups were mild and resolved with conservative treatment; no patient developed severe pancreatitis, organ failure, required ICU admission, or underwent invasive intervention. Therefore, the present data support comparable observed short-term adverse-event rates, but not definitive safety equivalence.

The differences in length of stay and cost also require restrained interpretation. Hospitalization cost was higher in the cholangioscopy-assisted group, consistent with additional device and disposable accessory costs. Total hospitalization cost in this study included all expenses from admission to discharge, including the electronic mother-baby cholangioscopy system and related consumables. Length of stay was shorter in the cholangioscopy-assisted group, but discharge timing can be influenced by institutional protocols, admission timing, post-procedure observation, nasobiliary-drain management, laboratory recovery, insurance processes, and bed-management factors. Therefore, the shorter stay should be viewed as an observed association rather than a direct causal effect of cholangioscopy.

Several limitations warrant consideration. First, this was a retrospective, single-center, non-randomized study. Although the conventional group was selected from contemporaneous cases using age-stratified random sampling and IPTW improved measured covariate balance, cholangioscopy use was still influenced by patient preference and the treating endoscopist’s clinical judgment. Selection bias and residual confounding by unmeasured factors therefore remain possible. Second, radiation assessment was limited to the RG1000 patient-position external dosimeter reading; fluoroscopy-system metrics, including fluoroscopy time, cumulative air kerma, and dose-area product, were unavailable. Third, the sample size limited the precision of adverse-event estimates and did not allow robust subgroup analyses. Fourth, follow-up was short, and the study could not evaluate long-term stone recurrence, re-intervention, or cost-effectiveness.

Future prospective, multicenter studies should define standardized indications for cholangioscopy-assisted ERCP, collect comprehensive radiation metrics, and include longer follow-up for recurrence and re-intervention. Formal cost-effectiveness analyses are also needed to clarify whether reduced radiation exposure and direct clearance confirmation justify the additional procedural time and device-related cost. In conclusion, cholangioscopy-assisted ERCP was associated with lower intra-procedural radiation-dose reading and fewer high-dose procedures in choledocholithiasis treatment. No statistically significant difference in short-term adverse events was observed, but longer procedure duration and higher hospitalization cost should be considered when selecting this approach.

## Data Availability

The raw data supporting the conclusions of this article will be made available by the authors, without undue reservation.
