# Supplementary material for: Cholangioscopy-assisted ERCP reduces radiation exposure in treating choledocholithiasis: a retrospective IPTW-adjusted cohort study
Source: Front Med (Lausanne). 2026 Jul 3;13:1816788. doi: 10.3389/fmed.2026.1816788 (PMC13375516; doi:10.3389/fmed.2026.1816788)
Supplement: Supplementary file 1 [file Table_1.docx]

**Supplementary Table S1. Covariate balance before and after IPTW**

| **Variable** | **Included in PS model** | **Unweighted SMD** | **IPTW-weighted SMD** |
| --- | --- | --- | --- |
| Age | Yes | 0.325 | -0.013 |
| Male sex | Yes | -0.080 | -0.023 |
| BMI | Yes | -0.048 | -0.049 |
| Hypertension | Yes | -0.040 | -0.019 |
| Diabetes | Yes | -0.186 | -0.055 |
| Coronary heart disease | Yes | -0.108 | -0.047 |
| Prior cholecystectomy | Yes | 0.267 | 0.023 |
| ≥2 stones | Yes | 0.285 | 0.045 |
| Maximum stone diameter | Yes | 0.306 | 0.029 |
| CBD diameter | Yes | 0.405 | 0.089 |
| Total bilirubin | Yes | -0.180 | -0.076 |
| Cholangitis | Yes | 0.042 | -0.002 |
| ASA score | Yes | 0.303 | -0.001 |

SMD, standardized mean difference; IPTW, inverse probability of treatment weighting; PS, propensity score. Absolute SMD <0.10 was considered adequate balance.
